# Insulin Resistance as a Risk Factor for Cutaneous Melanoma. A Case Control Study and Risk-Assessment Nomograms

**DOI:** 10.3389/fendo.2019.00757

**Published:** 2019-11-05

**Authors:** Alessandro Scoppola, Lidia Strigari, Agnese Barnabei, Pierpaolo Petasecca, Federica De Galitiis, Claudia Angela Maria Fulgenzi, Mario Roselli, Antonino De Lorenzo, Laura Di Renzo, Paolo Marchetti, Francesco Torino

**Affiliations:** ^1^Department of Oncology and Dermatological Oncology, Istituto Dermopatico dell'Immacolata-IRCCS, Rome, Italy; ^2^Department of Medical Physics, S. Orsola Malpighi University Hospital, Bologna, Italy; ^3^Endocrinology Unit, IRCCS Regina Elena National Cancer Institute, Rome, Italy; ^4^Department of Systems Medicine, Medical Oncology, University of Rome Tor Vergata, Rome, Italy; ^5^Section of Clinical Nutrition and Nutrigenomic, Department of Biomedicine and Prevention, University of Rome Tor Vergata, Rome, Italy; ^6^Department of Clinical and Molecular Medicine, Sapienza University of Rome, Rome, Italy

**Keywords:** insulin resistance, cutaneous melanoma, HOMA-IR, QUICKI, body mass index

## Abstract

Insulin resistance and obesity are suggested to have a key role in the molecular pathogenesis of various disorders, including several malignancies. Moreover, insulin resistance has recently been found to be associated with cutaneous and uveal melanoma, while a variable positive correlation between obesity and the risk of cutaneous melanoma was also found at least in men. The present trial aims at confirming whether insulin resistance, assessed with the homeostasis model assessment of insulin resistance (HOMA-IR) and the quantitative insulin sensitivity check index (QUICKI), is a risk factor for cutaneous melanoma. One hundred and thirty patients diagnosed with cutaneous melanoma and 130 age-, sex-, and skin phototype-matched controls were evaluated. At the univariate and multivariate analysis, the diagnosis of cutaneous melanoma was inversely related with insulin resistance (HOMA-IR) and positively with BMI (*p* = 0.0014 and *p* = 0.008, respectively). Consistently, insulin sensitivity (QUICKI) and BMI resulted positively associated with the diagnosis of cutaneous melanoma (*p* = 0.0001 and *p* = 0.0026, respectively). The results obtained are partially in agreement with those reported in the literature. By comparing our data with those generated by other studies, inconsistencies in key features among subgroups of different trials have emerged, possibly affecting final correlations. Based on insulin resistance/sensitivity, fasting insulinemia/glycemia, and BMI values collected from patients who participated in the present trial, two nomograms potentially assessing the risk of cutaneous melanoma have been generated. Molecular aspects sustain a role for insulin resistance in the carcinogenesis of cutaneous melanoma, but clinical data remain uncertain. Larger, well-balanced, correlative trials are still needed to define the potential role of insulin resistance in the carcinogenesis of cutaneous melanoma.

## Introduction

Over the last few decades, the incidence of cutaneous melanoma, the most commonly fatal form of skin cancer, has steadily increased worldwide (1). Incidence rates and increases in incidence rates vary significantly across populations of different ethnicities and geographical sites, and even within populations across age and gender (2). However, well-known risk factors for melanoma include skin type, personal history of previous melanoma, multiple clinically atypical moles or dysplastic nevi, a family history of melanoma and inherited genetic mutations (3). A possible association between dietary components and alcohol consumption and cutaneous melanoma has been found in some studies, but not in others (4–8). Moreover, an association with body mass index (BMI) (9, 10), waist-to-hip ratio (WHR), or height (11) has been suggested.

Insulin resistance (IR) identifies a disorder in which a given concentration of insulin is associated with a subnormal glucose response. IR seems to have a key role in the molecular pathogenesis of several different conditions, including type 2 diabetes mellitus, cardiovascular diseases, and certain malignancies associated with obesity, such as cancer of the esophagus (adenocarcinoma), colon, rectum, pancreas, gallbladder, liver, thyroid, kidney, prostate, breast (post-menopausal), ovary, and endometrium (12). More recently, IR has been also associated with cutaneous and uveal melanoma (13, 14). In meta-analyses of cohort and case-control studies, a variable positive correlation between obesity and risk of melanoma was found, at least in men (RR: 1.17–1.31) (15–18).

Several methods IR assessed by, including hyperinsulinaemic euglycaemic clamp (HEC) tests (the reference methods), and ratios derived from fasting insulin and glucose plasma levels, such as homeostasis model assessment of insulin resistance (HOMA-IR) (19) and the quantitative insulin sensitivity check index (QUICKI) (20, 21). In epidemiological studies HOMA-IR and QUICKI are more manageable when compared with gold standard tests, sharing good correlations (22). The present trial was aimed at defining whether IR, assessed by HOMA-IR and QUICKI, may be confirmed as a risk factor for cutaneous melanoma.

## Materials and Methods

### Study Population

The study evaluated adult patients with newly diagnosed, histologically confirmed cutaneous melanoma, referred to the Oncology Department of the Istituto Dermopatico dell'Immacolata (IDI-IRCSS) Hospital of Rome between April 2013 and January 2016. The disease was staged according to the Tumor Node Metastasis (TNM) staging system of the American Joint Committee on Cancer (AJCC) (7th Edition). Each case was matched for age, sex, and skin phototype to a healthy control. Controls were individuals of whom self-referred to the same hospital or to the Department of Nutrition of the Tor Vergata University of Rome. Patients and controls were eligible only if their personal history was free of cancer, hepatic, renal, or heart diseases, asthma, major hormonal disorders, autoimmune diseases, or chronic infection and complete blood count and electrolytes were normal. Information regarding sociodemographic data, lifestyle, medical history, anthropometric measurements and skin type (sensitivity to sun exposure) of participating individuals were also recorded by trained health professionals.

### Biochemical Analyses

Blood samples were drawn after an overnight fast, in order to measure circulating glucose and insulin levels. Samples were anonymized, blinded to the case–control status, centrifuged, and stored at −80°C. Average preservation time was similar for cases and controls. All samples were analyzed by technicians who were unaware of the hypothesis underlying the study. Human insulin levels were measured using radioimmunoassay assay (Millipore, Billerica, Massachusetts, USA) with a sensitivity of 2 mUI/ml for a 100 ml sample and inter-assay and intra-assay variability of 2.9–6.0 and 2.2–4.4%, respectively. Insulin resistance was assessed by calculating HOMA-IR values [HOMA-IR = fasting glucose (mg/dl) x fasting insulin (mU/l)/405] and QUICKI values [QUICKI = 1/log(I_0_) + log(G_0_)], where I_0_ is the fasting insulin (mUI/ml), and G_0_ is the fasting glucose (mg/dL) (20). The cut off value to define IR was HOMA-IR ≥ 2.50. Values typically associated with the QUICKI calculation for IR in humans fall broadly within a range between 0.45 for unusually healthy individuals and 0.30 in diabetics. Therefore, lower numbers reflect greater IR. The BMI was calculated and classified according to the recommendations outlined by the World Health Organization (WHO) (23). The local Ethics Committee approved the study protocol, and all participants provided a written informed consent.

### Statistical Analyses

Descriptive statistics (median, range, and percentages) of demographic, lifestyle, and anthropometric variables were calculated in each study participant. Student's *t*-test was used for comparing continuous variables between cases and controls, whereas χ^2^-test was used for categorical variables. The Pearson correlation coefficients were then calculated to study the association between insulin resistance expressed by HOMA-IR with age and anthropometric variables in healthy controls.

The possible association of HOMA-IR and QUICKI with melanoma incidence was examined by conducting regression analysis, controlling for namely skin type (categorical: sun sensitive and other), alcohol consumption (categorical: 0–11, 12–31, and >32 glasses/month), smoking status (categorical: yes/ex-smoker and non-smoker), BMI (ordered: with increment of one category more), etc. The same models were run with insulin alone or insulin controlling for glucose, as an alternative to HOMA-IR and QUICKI. A nomogram to predict melanoma risk was developed based on covariates retaining a statistically significant power (*P* < 0.05) in a multivariate analysis. To quantify the discrimination performance of the nomogram, Harrell's C-index was measured. Nomograms were subjected to bootstrapping validation (1,000 bootstrap resamples) to calculate a relatively adjusted C-index. Calibration was studied graphically after grouping patients into deciles according to their predicted probabilities and plotting the mean predicted probabilities against the mean observed probabilities. To compare findings of our study cohort with those reported in similar published studies two-way ANOVA and Tukey's multiple comparisons *post-hoc* tests were applied. Statistical analysis was performed using the computing environment R (24).

## Results

One hundred and thirty patients diagnosed with cutaneous melanoma and 130 age-, sex-, and skin phototype matched controls were evaluated. The characteristics of the study participants are reported in Table 1.

**Table 1:** Characteristics of the study participants.

	**Melanoma group**	**Control group**	**Statistic significance**
Total	130	130	
**Age (years)**			
Median [range]	51.5 [18–86]		Matched variable
**Sex**			
Female/Male	72/58		Matched variable
**Phototype**			
I	5 (4%)		Matched variable
II	44 (34%)		
III	51 (39%)		
IV	27 (21%)		
V	3 (2%)		
VI	0 (0%)		
**Stage**			
IA	119	–	
IB	6	–	
IC	2	–	
IIA	1	–	
IIIB	1	–	
IIIC	1	–	
**Smoking**			
No	52 (39.8%)	46 (35.7%)	*P* = 0.59
Yes/ex-smoker	78 (60.2%)	84 (64.3%)	
**Alcohol consumption (glasses per month)**			
0–11	24 (18.8%)	21 (16.1%)	
12–31	84 (64.9%)	92 (70.5%)	*P* = 0.73
32+	22 (16.3%)	17 (13.4%)	
**BMI (Kg/m**^**2**^**)**			
Median (range)	24.9 (18.8–36.0)	24.2 (18.7–41.1)	*P* = 0.12
**BMI subgroups**			
<25	67 (51.5%)	81 (62.3%)	
≥25 to <30	36 (27.7%)	36 (27.7%)	*P* = 0.03
30+	27 (20.8%)	13 (10.0%)	
**Fasting insulinemia (mUI/ml)**			
Median (range)	6.8 (0.15–26)	8.9 (1.4–36.4)	*P* = 0.0006
**Fasting glycaemia (mg/dl)**			
Median (range)	96 (74–245)	94 (53–115)	*P* = 0.003
**Fasting glycaemia (nmol/L)**			
Median (range)	5.28 (4.1–13.5)	5.17 (2.9–6.3)	*P* = 0.003
**HOMA-IR (adm)**			
Median (range)	1.64 (0.07–6.48)	1.94 (0.3–8.9)	*P* = 0.015
**QUICKY (adm)**			
Median (range)	0.35 (0.29–0.70)	0.34 (0.28–0.48)	*P* = 0.0018

*Adm, adimensional value*.

Among the case group, disease was diagnosed in 119 patients (91.5%) at stage Ia, in 6 (4.6%) patients at stage Ib, 2 (1.5%) patients at stage Ic, in 1 (0.8%) patient at stage IIa, in 1 (0.8%) patient at stage IIIb, and in 1 (0.8%) patient at stage IIIc.

We found a statistically significant difference in fasting insulinaemia, glycaemia, HOMA-IR, and QUICKI between the group of patients with melanoma and the control group (Table 1). In contrast, no difference was found in BMI values between the two groups (*p* = 0.12).

The HOMA-IR median value was 1.64 (0.07–6.48) in the case group and 1.94 0.3–8.9) in the control group (*P* = 0.015). The < QUICKI median value was 0.35 (0.29–0.70) in the case group and 0.34 (0.28–0.48) in the control group (*P* = 0.0018).

HOMA-IR was positively correlated with a BMI both in the melanoma (r = 0.49, *p* < 0.001) and in the control group (*r* = 0.32, *p* < 0.001). Similarly, QUICKI showed a negative correlation with a BMI both in the melanoma (r = −0.39, *p* < 0.001) and in the control group (*r* = −0.28, *p* < 0.001).

In the case group, the median value of HOMA-IR was 1.31 (0.36–4.58) in patients with a BMI <25, 1.89 (0.07–6.48) in patients with a BMI ≥25 and <29, 2.68 (0.63–5.82) in patients with a BMI ≥30.

In the control group, the median value of HOMA-IR was 1.89 (0.3–8.1) in individuals with a BMI <25, 1.95 (0.68–5.14) in those with a BMI ≥25 and <29 and 3.16 (1.245–8.91) in those with a BMI ≥30.

In the case group, the median value of QUICKI was 0.37 (0.30–0.46) in patients with a BMI <25, 0.35 (0.29–0.70) in patients with a BMI ≥25, and <29, 0.33 (0.30–0.41) in patients with a BMI ≥30.

In the control group, the median value (range) of QUICKI was 0.35 (0.28–0.48) in individuals with a BMI <25, 0.34 (0.30–0.41) in individuals with a BMI ≥25 and <29, 0.33 (0.30–0.41) in individuals with a BMI ≥30.

In the whole population, HOMA-IR resulted to be correlated with insulinemia (r = 0.96, *p* < 0.0001), glycaemia (*r* = 0.21, *P* = 0.0008), weight (*r* = 0.35, *p* < 0.0001), sex (χ test, *p* = 0.01), but not with age (*r* = 0.08, *p* = 0.17), and height (*r* = 0.03, *p* = 0.58).

In the whole population, QUICKI resulted to be inversely correlated with insulinemia (*r* = −0.76, *p* < 0.0001), weight (*r* = −0.31, *p* < 0.0001), but not with glycaemia (*r* = −0.07, *P* = 0.25), sex (χ^2^ test, *p* = 0.63), age (r = 0.03, *p* = 0.60), and height (*r* = −0.06, *p* = 0.36).

At the univariate and multivariate analysis (Tables 2, 3), HOMA-IR was inversely associated with the diagnosis of cutaneous melanoma, while BMI resulted positively associated with diagnosed malignancy (*p* = 0.0014 and *p* = 0.008, respectively). Consistently, QUICKI and BMI resulted positively associated with the diagnosis of cutaneous melanoma (*p* = 0.0001 and *p* = 0.0026, respectively).

**Table 2:** Univariate analysis.

	**Coefficient**	**Std. error**	***P*-value**
**Model 1**			
BMI (Kg/m^2^)	0.0506	0.0328	0.1228
QUICKY (adm)	11.6227	3.8252	0.0024
HOMA-IR index (adm)	−0.2459	0.1040	0.0180
Fasting insulinemia (mUI/ml)	−0.0883	0.0269	0.0010
Fasting glycaemia (mg/dl)	0.0297	0.0105	0.0046
Fasting glycaemia (mmol/L)	0.0297	0.0105	0.0046

**Table 3:** Multivariate analysis of melanoma risk and model Harrell's C-index (i.e., area under curve, AUC).

	**Coefficient**	**Std. error**	***P*-value**
**Model 1 (AUC** **=** **0.8485)**			
QUICKY (adm)	20.9814	4.7165	<0.0001
Fasting glycaemia (nmol/L)	0.0425	0.0129	0.0010
BMI (Kg/m^2^)	0.0743	0.0393	0.0589
**Model 2 (AUC** **=** **0.8475)**			
HOMA-IR (adm)	1.2987	0.4884	0.0078
Fasting insulinemia (mUI/ml)	−0.4217	0.1215	0.0005
BMI (Kg/m^2^)	0.0648	0.0391	0.0975

**Figure 1:**
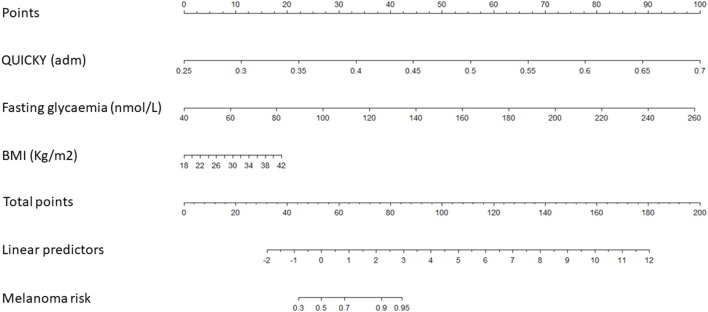
A risk-assessment nomogram based on Model 1.

**Figure 2:**
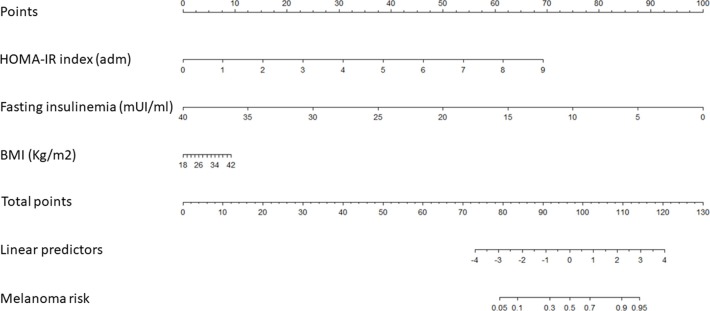
A risk-assessment nomogram based on Model 2.

Based on the estimated regression coefficients of models 1 and 2, two nomograms were developed to estimate the risk of melanoma in our cohort (Table 3, and Figures 1, 2). The C-index for models 1 and 2 were 0.8485 and 0.8475, respectively. Calibration of nomograms was considered adequate.

Model 1 indicates that higher incidence of melanoma is observed when patients have higher QUICKY values, fasting glycaemia and BMI. Model 2 indicates that higher incidence of melanoma is observed when patients have higher HOMA-IR values and BMI and lower fasting insulinemia.

**Figure 3:**
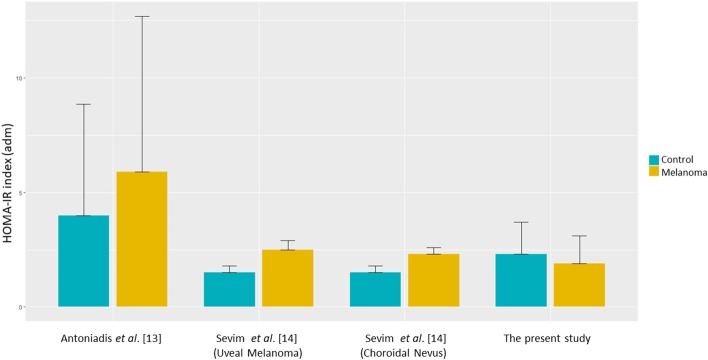
HOMA-IR values in patient vs. control subgroups among the avalaible studies.

Finally, as our HOMA-IR values appeared to be inconsistent when compared to those reported in other studies [i.e., (13, 14)], we used the two-way ANOVA and Tukey's multiple comparisons *post-hoc* tests to compare the values of HOMA-IR in the melanoma and in the control group reported in those studies. Based on those analyses, a statistically significant difference was found between Antoniadis et al. findings (13) and those obtained in Sevim et al. (14) and in our study cohorts (Figure 3).

## Discussion

The incidence and mortality rates of cutaneous melanoma are still on the rise (25). While its incidence is >10 times lower than that of other skin cancers (26), its capacity to rapidly metastasize and affect younger patients makes the malignancy a significant health and socio-economic burden (26–28). Although current clinical, biochemical, and histological methods provide insights into disease behavior and outcome, melanoma is still an unpredictable disease (2). Therefore, significant efforts are still needed in seeking new risk factors that could improve early diagnosis and current preventive measures. Obesity in men and IR in both sexes have been suggested as potential risk factors for several malignancies, including cutaneous melanoma (12, 15–18). More recently, IR assessed with HOMA-IR was correlated with uveal melanoma and atypical nevi (14).

The present case-control study investigated IR, assessed by HOMA-IR, and QUICKI, as a risk factor for cutaneous melanoma. We used HOMA-IR because it is a simple, validated, sensitive and specific measure of IR, which holds good correlation with the gold standard HEC tests (22). Furthermore, we assessed IR by the QUICKI, as the log-transformation contained within its formula, in comparison with HOMA-IR results with greater accuracy and stronger correlation with the HEC tests, therefore providing an improvement estimating IR (20, 29). The limitations of HOMA-IR and QUICKI in assessing IR, include the fact that these tests cannot provide information on the activity of insulin receptors (22).

In our study population consisting of 260 individuals (130 patients affected by non-metastatic cutaneous melanoma and 130 age-, sex-, and skin phototype-matched controls), a statistically significant difference was found in fasting insulinaemia, glycaemia, HOMA-IR, and QUICKI between the two groups, but not in the other variables considered, including BMI. A strong correlation was found between HOMA-IR and blood insulin levels (Pearson test: *r* = 0.97, *p* < 0.0001) and, at a lesser strength, between HOMA-IR and weight (*r* = 0.35, *p* < 0.0001), glycaemia (*r* = 0.21, *P* = 0.0008), and sex (χ^2^ test, *p* = 0.01), but not with age (*r* = 0.08, *p* = 0.17) nor height (*r* = 0.03, *p* = 0.58). Similarly, QUICKY values were strongly inversely correlated with insulinaemia (*r* = −0.76, *p* < 0.0001) and weight (*r* = −0.31, *p* < 0.0001), but not with the other variables considered. Moreover, HOMA-IR values correlated with BMI in both the group of patients (*r* = 0.49, *p* < 0.001) and controls (*r* = −0.28, *p* < 0.001). Consistently, QUICKY showed a negative correlation with BMI both in the case (*r* = −0.39, *p* < 0.001) and control groups (*r* = −0.28, *p* < 0.001). Overall, these findings confirm the reliability of the two tests and therefore their interchangeability in assessing IR.

However, at the univariate and multivariate analysis, IR evaluated with HOMA-IR and QUICKI, did not result to be a risk factor for cutaneous melanoma. Indeed, the HOMA-IR median value was 1.64 (0.07–6.48) in the group of patients and 1.94 (0.3–8.9) in the control group (*p* = 0.015). Similar results were obtained by QUICKI (*P* = 0.0018). Importantly, when the HOMA-IR value was divided into quartiles, in the groups of individuals affected by melanoma, only few patients had HOMA-IR values within the range of the quartile containing the highest values, being normally distributed in the control group. This aspect needs to be taken into consideration in order to correctly interpret the correlative results. Our findings are not consistent with those reported by Antoniadis et al. (13) in the only available case-control study that evaluated the correlation between IR assessed by HOMA-IR in a cohort of 55 patients affected by cutaneous melanoma (stage I-IV) compared with 165 controls. The Authors showed that IR correlated with the diagnosis of cutaneous melanoma. However, several findings differ between individuals of our population and those enrolled in the study of Antoniadis et al. (13) (Figure 3). First, in that study, patients affected by cutaneous melanoma had higher HOMA-IR mean levels (almost 50% higher; *p* = 0.05) compared to those measured in the control group. In our study population, median values of HOMA-IR were significantly lower in the case group compared with the control group. Moreover, HOMA-IR values registered in our case group were lower, compared with those reported in the homologous group evaluated by Antoniadis et al. (13). This may be presumably attributed to diet and lifestyle choices between the two cohorts, belonging to different populations (Italian and Greek, respectively) or to unknown factors. In addition, genetic differences cannot be excluded. Large epidemiological studies aimed at defining cut-off values of glycaemia, insulinaemia, and IR in each population might be useful in highlighting the differences between the two populations. Unfortunately, data are still lacking and therefore, no comparisons (even indirect) can be made. Secondly, we found a potentially confounding difference between the two studies regarding the balance between females and males. In our cohort, females were 56% compared to 42% in the study of Antoniadis et al. (13). These aspects could have influenced the final findings. Indeed, it has been reported that women have a lower risk of developing skin cancer, including melanoma, presumably related to less sun exposure, especially in the subgroup of women with a higher BMI (Reviewed in 18). Moreover, although the assessment of IR by HOMA-IR was suggested to be corrected according to weight and/or sex (30), the formula of HOMA-IR remains unmodified in its current use.

Finally, in Antoniadis et al. study (13), ~50% of patients had stage III disease (and another 16% of patients with a stage IV disease), while in our cohort of patients the vast majority of cutaneous melanoma was diagnosed at an earlier stage (97.7% at stage I). These findings suggest that, in melanoma patients, higher levels of IR are related to higher stage of disease. Therefore, higher levels of IR could be found more probably in later phases of the disease. Possibly, at an earlier stage, the contribution of IR, as “initiating factor”, might be difficult to differentiate from other confounding risk factors, e.g., UV exposure. Alternatively, unbalances of the insulin/IGF-1/IGF-2 signaling pathways might be more important for the progression of the disease in its later stages. However, data on prognosis and survival rates for patients with melanoma as related to IR are still lacking and such correlations need to be elucidated in further trials.

Recently, the role of IR as risk factor for another malignancy arising from pigmented cells, namely uveal melanoma, was evaluated by Sevim et al. (14) in a retrospective trial of 86 patients, in comparison to 38 patients with choroidal nevus, and 86 controls. They found significantly higher HOMA-IR values in patients affected by uveal melanoma compared to those with choroidal nevi and the control group (2.5 ± 0.4, vs. 2.3 ± 0.3 vs. 1.5 ± 0.3, respectively; *P* < 0.001), suggesting that IR may increase the risk of uveal melanoma. Moreover, in Sevim et al. study (14), fasting glycaemia and basal insulin levels were found to be significantly higher in the uveal melanoma group compared with controls (*p* < 0.005 and 0.001, respectively), as like as in Antoniadis et al. study (13), but not in our study population (Figure 3).

As our study prospectively evaluated IR in a large cohort of patients diagnosed with cutaneous melanoma and age-, sex-, and skin phototype-matched controls, we thought of developing two nomograms aimed at evaluating the personal risk of cutaneous melanoma in single individuals based on HOMA-IR/QUICKI values, fasting insulinemia/glycemia and BMI (C-index for models 1 and 2 was 0.8485 and 0.8475, respectively).

In nomogram 1, based on the underlying model 1, values of QUICKY, fasting glycaemia and BMI are used as input variables predicting the risk of developing melanoma. A higher risk of melanoma is observed when individuals show higher QUICKY, fasting glycaemia and BMI values. In nomogram 2, according to model 2, HOMA-IR, fasting insulinemia and BMI values were used as input variables predicting the risk of developing melanoma. In this model, higher risk of melanoma is observed when individuals show higher HOMA-IR values and BMI and lower fasting insulinemia. Calibration of both nomograms was evaluated and appeared adequate. These data highlight that values of IR could be useful in predicting one's personal risk of cutaneous melanoma. However, before its use is applied in clinical practice, nomograms need to be validated in larger prospective correlative trials.

To our knowledge, we report the findings of the largest prospective case-control study (with the largest cohort of melanoma patients) evaluating the correlation between IR and cutaneous melanoma. Moreover, it is the first study providing nomograms aimed at exploiting information related to IR to define the personal risk of cutaneous melanoma, based on IR assessment and BMI. The study was conducted in accordance with the most up-to-date technical methodologies and most accurate statistical evaluations allowing to highlight any potential bias in establishing correlations between IR and diagnosis of cutaneous melanoma. Furthermore, the quality of data allowed the creation of two nomograms, which may represent tools that are potentially capable of predicting the risk of developing cutaneous melanoma, once validated. Limitations of the study include the sample size of the study population and potential bias related to the recruitment of cases and controls, and those who were self-referred only to two hospitals within a limited geographical area. Moreover, participants were only Caucasians. Therefore, our findings might not be generalized to individuals or populations of different ethnicities. Finally, dual-energy X-ray absorptiometry (DXA) was not assessed, nor vitamin D levels. Similarly, the assessment of other hormones related to obesity and in turn to IR was not among the objectives of the study and remains to be explored.

Overall, the results of the present study seemed to not directly confirm IR as a risk factor for cutaneous melanoma. However, our findings need to be prudently interpreted as the imbalance of IR values between the case and control group could have biased results. Nevertheless, when data were exploited to create nomograms capable of defining the personal risk of cutaneous melanoma, IR's role as a risk factor for the disease has emerged. The suggested nomograms are however immature for their clinical use in clinical practice, as they need to be validated in large prospective clinical trials.

## Conclusions

Despite molecular aspects do prompt us to consider IR as having an important role in the carcinogenesis of cutaneous melanoma, clinical data still do not confirm the hypothesis. Our findings highlight that any uncertainty in considering IR as a risk factor for cutaneous melanoma can be attributed to the selection of individuals of study populations. Larger, well-balanced, correlative trials are still needed to define the potential role of IR in the carcinogenesis of cutaneous melanoma.

## Data Availability Statement

The datasets generated for this study will not be made publicly available due to privacy policy.

## Ethics Statement

The studies involving human participants were reviewed and approved by Comitato Etico IDI-IRCCS, Rome. The patients/participants provided their written informed consent to participate in this study.

## Author Contributions

AS, PM, and FT contributed conception and design of the study. PP, AB, LD, and CF collected data and organized the database. LS, AB, and FT performed the statistical analysis and wrote the first draft of the manuscript. AD, MR, PM, AS, and LD supervised the research. All authors contributed to manuscript revision, read, and approved the submitted version.

### Conflict of Interest

The authors declare that the research was conducted in the absence of any commercial or financial relationships that could be construed as a potential conflict of interest.
